# White Hop Shoot Production in Slovenia: Total Phenolic, Microelement and Pesticide Residue Content in Five Commercial Cultivars

**DOI:** 10.17113/ftb.57.04.19.6269

**Published:** 2019-12

**Authors:** Mateja Vidmar, Veronika Abram, Barbara Čeh, Lea Demšar, Nataša Poklar Ulrih

**Affiliations:** 1 Department of Food Science and Technology, Biotechnical Faculty, University of Ljubljana, Jamnikarjeva 101, SI-1111 Ljubljana, Slovenia; 2 Slovenian Institute of Hop Research and Brewing, Cesta Žalskega tabora 2, SI-3310 Žalec, Slovenia

**Keywords:** white hop shoots, hop cultivars, phenolics, antioxidant potential, microelements, active ingredients of pesticides

## Abstract

Harvesting of white hop shoots might be justified if they can be shown to be beneficial to human health. The aim of the present study is to determine the effects of hop cultivars and year of production on total phenolics, antioxidant potential, microelements and pesticide residues. Biomass per plant was highly variable across the cultivars (3.1-7.1 g dry mass per plant) and depended on hop cultivar and year (2009-2011). Total phenolics as chlorogenic acid equivalents (CAE) on dry mass basis varied from 0.60 to 1.80 mg/g, and showed significant effects across hop cultivar and year. The radical scavenging activities of the samples collected in years 2010-2012 ranged from 11 to 19 µg CAE. Ferric reducing activity was <0.01, with significantly different effects across hop cultivars (p_C_≤0.05) and year (p_y_≤0.05) observed only in 2012. Traces of microelements and potentially active compounds from the use of pesticides in white hop shoots of *Humulus lupulus* ‘Dana’ were analysed. The content of zinc in the hop shoots on dry mass basis was very low (4 mg/kg), and it was below the limit of detection in the soil. The content of copper in the hop shoots was also very low (2.3 mg/kg), while in the soil it was below the critical emission (100 *vs* 300 mg/kg, respectively). All 182 active ingredients from the residues of the previously used pesticides were below the limits of detection. It can be concluded that these white hop shoots are better antioxidants than hop cones and hop leaves, and that they do not contain any pesticide residues.

## INTRODUCTION

Hop (*Humulus lupulus* L.; Cannabinaceae) is a perennial plant that is cultivated almost exclusively for its secondary metabolites. Apart from the well-known alpha and beta acids in the hop cones that are used in the production of beer for their characteristic bitterness and aroma, other hop components are receiving more attention as antioxidants and potential antibacterial, antiviral and anticancer agents (*1*, *2*).

In culinary circles, the young spring shoots are eaten like asparagus (*3*). Early in the spring, from 15 to 40 buds appear on the hop root systems. These grow rapidly and develop into the hop shoots. When they emerge from the soil, they become green. Only four to ten shoots per plant are then used for hop growing, and the rest are removed as waste. However, when they are approx. 30 cm long, they can be regarded as the most expensive vegetable in the world. Their high price results from the few days available for their collection, and their laborious harvest (*3*).

The white hop shoots are collected before they emerge from the soil. At this stage, they are still fragile and are less bitter and not tough, but they have to be dug out of the soil manually. Traditionally, in Slovenia this is performed before the regular agrotechnical procedure for pruning (cutting) of the top of the hop root system, which is carried out in April.

The world hop production has been in decline for several years, although only recently an increase has been observed (*4*). The production of hops in Slovenia covers 1667 ha and represents about 3% of the global hop area. According to the same source, hop production for 2018 was 3078 million tonnes (*4*).

Harvesting of white hop shoots might be an opportunity for small hop growers if these shoots can be shown to be beneficial to human health. *In vitro* investigations of hop cones and hop leaves have demonstrated the antioxidant and antimicrobial potential of phenolics extracted from five hop cultivars grown in Slovenia, Austria, Germany and the Czech Republic (*1*). To date, to the best of our knowledge, the literature contains only one study about the phenolic content and antioxidant potential of the wild hop shoots (*5*). On the other hand, concerns over the use of white hop shoots for culinary purposes have arisen, because people are not familiar with the pesticides used in hop fields. The main concern is a possible high content of heavy metals and potentially active compounds derived from the pesticides.

The aim of the present study is thus to determine the effects of weather conditions on the yield of white hop shoots of five hop cultivars: *H. lupulus* ‘Aurora’, ‘Celeia’, ‘Dana’, ‘Hallertauer Magnum’ and ‘Savinjski golding’ collected from 2009 to 2011 early in the spring (*i.e*. before their emergence). White hop shoots collected from 2010 to 2012 were extracted with ethanol to determine their total phenolics and antioxidant potential. Microelements copper and zinc, and potentially active compounds that might have arisen from the use of pesticides were analysed in samples of *H. lupulus* ‘Dana’ shoots collected in 2010 only, along with the soil from the same hop-growing location.

## MATERIALS AND METHODS

### Plant materials and collection

Table 1 (*6*) gives details of the origin, harvest times and brewing purpose of the selected five hop cultivars: *Humulus lupulus* ‘Aurora’, ‘Celeia', ‘Dana’, ‘Hallertauer Magnum’ and ‘Savinjski golding’. The white hop shoots were collected in the first half of April in 2009, 2010, 2011 and 2012 just before their emergence, and before the regular agrotechnical pruning of the top of the root system. Initially, in 2009, the white hop shoots of each of the five cultivars were dug out of the soil from around three plants, and then in 2010, 2011 and 2012 samples were taken from 15 to 20 plants of each cultivar. Consecutive plants were selected from a central row in each hop field. Plants from the edge of the field were omitted. All of the hop fields were in their fully productive period in each year of sampling. One exception has to be stressed here. Only the shoots of *H. lupulus* ‘Dana’ were more developed than the shoots of other cultivars on the collection date in 2011 (16 April). Their green tops were just coming out of the soil. Nevertheless, we dug away the soil and the shoots of *H. lupulus* ‘Dana’ were cut from the root system in the same way as those of the other cultivars because they were still white in the lower part.

**Table 1:** Background details of the hop cultivars used in the present study (*6*)

Cultivar	Origin	Maturity class	Primary purpose in brewing
‘Aurora’	Slovenia	Medium early	Aroma
‘Celeia’	Slovenia	Medium late	Aroma
‘Dana’	Slovenia	Medium early	Bittering and aroma
‘Hallertauer Magnum’	Germany	Medium early	Bittering
‘Savinjski golding’	Slovenia	Early	Aroma

Each plant root system had from 15 to 40 white shoots, with the mean fresh mass of each shoot of approx. 1 g. Immediately after the harvest, the white hop shoots of each plant were washed, dried on paper, and weighed, with a sample taken for moisture content. Then all of the samples within each cultivar were combined. Each annual sample per cultivar was divided into two halves: one half was dried at 35 to 40 °C to constant mass, and the other half was frozen and stored in a freezer at  -20 °C for later analysis. The mass of dry white hop shoots was determined using the samples from 2009-2011, and chemical analyses were done on samples from 2010-2012. In 2010, *H. lupulus* ‘Dana’ shoots and the soil from the same growing location were additionally collected to determine the contents of copper, zinc and active compounds of pesticides.

### Location characteristics and weather conditions

Since two cultivars were grown in the same field (*H. lupulus* ‘Dana’ and ‘Hallertauer Magnum’), four fields were selected for sampling, located in Žalec, Savinja Valley (Slovenia), a traditional hop-growing region, on medium deep eutric brown soil on a sandy gravel base. The soil texture of the upper layers was classified as the clay loam to sandy clay loam (*i.e*. medium to heavy soil).

In 2010, soil samples were collected from each of the hop fields (approx. 0.5 kg from each hop field, taken from 20 to 25 places with the soil sampling probe to a depth of 0 to 25 cm, going zig-zag across each hop field). Soil sampling probe was Auger for arable land, Ø 13 mm, operational length 25 cm, total length 58 cm, graduation 5 cm, totally zinc-plated construction (Eijkelkamp Soil & Water, Giesbeek, The Netherlands). Soil samples were frozen and stored in a freezer at -20 °C for chemical analysis. Quantitative determination of soil texture, mechanical analysis, was done according to the standard sedimentation method with particle size distribution in a combination of sieving and sedimentation technique (*7*). pH was determined in 1 M potassium chloride solution; P_2_O_5_ and K_2_O were analysed in the ammonium lactate (AL method; pH=3.7) extract of the soil and/or spectrophotometric determination with ammonium molybdate (phosphor) and with atomic absorption spectroscopy (potassium) (*8*, *9*). Magnesium was analysed with the CaCl_2_ method (*10*), and humus content with the ISO 14235:1998 method (*11*). Table 2 gives the data from the initial analysis of the soil characteristics, along with the soil supply classes for the elements (A=very low to E=excessive). These data indicate a well-supplied soil, where the phosphate (P_2_O_5_), potassium (K_2_O) and magnesium levels generally tended to be high (ranges: 24.1-40.2, 28.0-34.8 and 5.6-39.3 mg/100 g, respectively). All of the hop fields had good humus content (overall mean 3.0%), with mean pH=6.8.

**Table 2:** Chemical characteristics of the soil from the fields where the different hop shoot cultivars were sampled

Parameter	Cultivar			
‘Aurora’	‘Celeia’	‘Dana’ and ‘Hallertauer Magnum’	‘Savinjski golding’
pH	7.2	6.6	6.7	6.6
*w*(P_2_O_5_)/(mg/100 g)	28.0	40.2	24.1	39.0
Soil supply class (P_2_O_5_)	D	E	C	D
*w*(K_2_O)/(mg/100 g)	34.6	28.0	34.8	30.0
Soil supply class (K_2_O)	D	C	D	C/D
*w*(Mg)/(mg/100 g)	39.3	-	15.8	5.6
Soil supply class (Mg)	E	-	D	C
*w*(humus)/%	3.7	2.6	2.6	3.1
Humus content	C	C	C	C

A=very low, B=low, C=good, D=oversupplied (P_2_O_5_ between 26 and 40 mg/100 g), E=excessive (P_2_O_5_˃40 mg/100 g)

The soil where *H. lupulus* ‘Dana’ hop shoots were grown was taken separately as described above to analyse the microelements copper and zinc, and pesticide residues, along with the respective hop shoots.

**Fig. 1:**
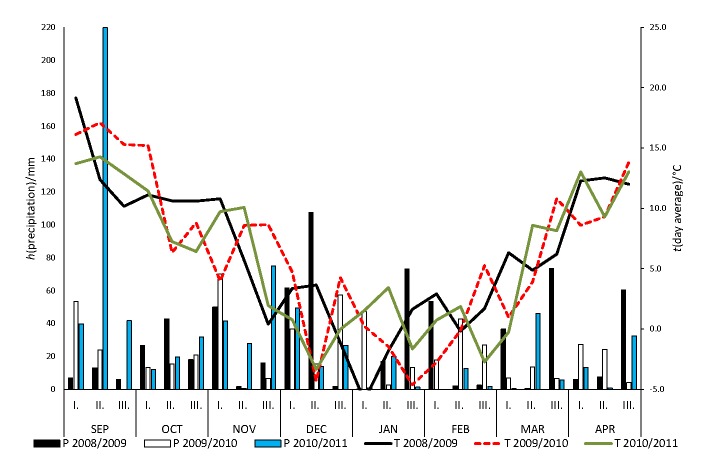
Precipitation (P) and mean daily temperatures (T) per 10-day periods from September to April in the years 2009, 2010 and 2011. I=the first ten days of the month, II=the second ten days of the month, and III=the third the days of the month

The weather during the winter and spring (the period between the growing seasons) is important for hop, especially for its root hibernation because it is a perennial plant. Therefore, Fig. 1 shows the weather conditions, such as precipitation per 10-day periods and mean 10-day period temperatures, after the hop harvest of the previous years (beginning of September) to April (the month of the white hop shoot collection) in years 2009, 2010 and 2011. These data were obtained from the Adcon Meteorological Station (Slovenian Institute of Hop Research and Brewing (IHPS), Žalec, Slovenia). The lowest precipitation in these periods was from autumn 2009 to spring 2010 (548 mm), while from autumn 2008 to spring 2009 and in 2010/2011, the precipitation was higher (685 and 735 mm, respectively). It is worth mentioning that there was an extraordinary precipitation level in the second 10-day period in September 2010, reaching 220 mm in 10 days, which resulted in high levels of run-off and had a strong impact on the total precipitation for the autumn to spring 2010/2011 period. Because of that 10-day period, the precipitation in the interval from autumn 2010 to spring 2011 was higher than in the same period between 2009 and 2010. The overall mean daily temperature during these 8 months in 2008/2009 and 2009/2010 was 6.2 °C, and in 2010/2011 it was 5.8 °C. From February to the first half of April (*i.e.* to the time of white hop shoot collection), the highest precipitation was in 2009 (182 mm), which also saw the highest mean temperature (5.8 °C) followed by 2010 (167 mm) and 2011 (81 mm), with mean temperatures for this period of 4.5 and 4.8 °C, respectively.

### Chemicals

Folin-Ciocalteu reagent, chlorogenic acid and 2,2-diphenyl-1-picrylhydrazyl (DPPH) were from Fluka (Buchs, Switzerland). Potassium hexacyanoferrate(II) was from Kemika (Zagreb, Croatia), and iron(III) chloride was from Carlo Erba (Cornaredo, Italy). All other chemicals and solvents were of analytical purity.

### Moisture content

The moisture content of fresh white hop shoots (from 2009-2011) was analysed according to the method SIST EN ISO 665:2001 (*12*). The samples (5 g of each cultivar) were weighed in an aluminium pan and dried at 102-104 °C for 3 h (VO400; Memmert GmbH+Co.KG, Schwabach, Germany). The drying was repeated at least once until constant mass, with all samples analysed in parallel duplicates.

### Extraction of phenolics

The frozen white hop shoot samples of all five cultivars from the years 2010, 2011 and 2012 were thawed at room temperature, shredded (5 g) into the extracting solvent (20 mL 96% ethanol) and mixed at 60 °C for 24 h in a water bath (Kambič, Semič, Slovenia). Once cooled, the suspensions were centrifuged at 3600×*g* for 10 min (Centric 322B centrifuge; Tehtnica/Domel, Železniki, Slovenia), the supernatants were collected and used for immediate determination of total phenolics. The rest of the supernatants were frozen to -20 °C and later used for the antioxidant potential assays. In each extraction, two parallel samples of each cultivar were extracted.

### Total phenolics

Total phenolics were determined by the method of Singleton and Rossi (*13*). A suitable volume of each extract was diluted with deionised water to 2.75 mL. Following the dilution of the Folin-Ciocalteu reagent with deionized water (1:1), 0.5 mL of this was added to the diluted extracts, and after exactly 5 min, 0.5 mL 20% Na_2_CO_3_ was added. These samples were left for 90 min at room temperature, and then their absorbance was read against a blank (96% ethanol) at 746 nm in a spectrophotometer (HP DAD UV/Vis 8453; Hewlett Packard, Agilent Technologies, Santa Clara, CA, USA). All supernatants were analysed in three parallel batches, and total phenolics were expressed in mg chlorogenic acid equivalents (CAE) per mL extract. For this purpose, a calibration curve was constructed in the range from 0 to 80 µg chlorogenic acid and dissolved in 96% ethanol, which provided a linear correlation (y=0.0181x) with a correlation coefficient R^2^=0.996.

### DPPH antioxidant potential

The 2,2-diphenyl-1-picrylhydrazyl radical (DPPH**˙**) scavenging assay was used to determine the antioxidant potential of the obtained extracts. DPPH is characterised as the stable free DPPH**˙**. When the violet-coloured solution of DPPH**˙** is mixed with an antioxidant that can donate an electron or a hydrogen atom, this gives rise to the reduced form of DPPH**˙.** This reduction in DPPH**˙** can be followed by monitoring the decrease in the absorbance during the reaction (*14*). The samples were prepared with 750 µL 96% ethanol, 250 µL suitably diluted extract, and 250 µL 0.51 mM DPPH in 96% ethanol. The control samples contained only 1 mL 96% ethanol and 250 µL 0.51 mM DPPH in 96% ethanol. Every sample was mixed well, and after 15 min the absorbance was read at 517 nm. The data is expressed as IC_50_ value, which was calculated from the concentration of phenolics needed to reduce the absorbance (*A*) value by 50% using the following equation:

IC_50_=(*γ*_P_*/*R)*·V*_sup_ /1/

where *γ*_P_ is the mass concentration of the phenolic compounds as CAE (mg/mL), R is the dilution factor of the extract necessary for 50% inhibition of DPPH**˙**, and *V*_sup_ is the volume of the supernatant from the sample (as indicated in the DPPH**˙** scavenging activity assay).

### Ferric reducing antioxidant power

For the ferric reducing antioxidant power (FRAP) assay, the method of Juntachote *et al.* (*15*) was used. Here, 2.5 mL 50 mM phosphate buffer, pH=6.8, were pipetted into six test tubes and up to 0.5 mL 96% ethanol (every 0.1 mL) were added. After short vortexing (MS3 Basic vortexer; IKA, Staufen, Germany), up to 0.5 mL extracts were added, followed by 2.5 mL 1% potassium hexacyanoferrate(II) and 2.5 mL 20% trichloroacetic acid. The samples were mixed again and centrifuged at 15800×*g* for 15 min (microcentrifuge 5415C; Eppendorf, Hamburg, Germany). From the obtained supernatants 2.5 mL were transferred to new test tubes. These supernatants were diluted with 2.5 mL water, and 1 mL 1% iron(III) chloride was added. The reaction mixture was vortexed, and after 25 min in the dark the absorbance was measured at 740 nm with a spectrophotometer (HP DAD UV/Vis 8453; Hewlett Packard, Agilent Technologies). The final data were defined from the mean value of the slope of the straight line (m) for the correlation between the absorbance and the concentration of the phenolics in the final reaction mixture (*15*), as calculated using the following equation:

*γ*(TP)_f_*=γ*(TP)_b_*·V*_sup_*·*2.5*/*R /2/

where *γ*(TP)_f_ is the final total phenolic concentration, *γ*(TP)_b_ is the total phenolic concentration at the beginning of the assay, *V*_sup_ is the volume of the supernatant of the tested extract, and R is the dilution factor.

### Microelements and potentially active compounds from pesticide residues

Microelements (copper and zinc) and potentially active compounds from pesticide residues in the frozen samples of *H. lupulus* ‘Dana’ shoots and the soil from the same cultivation location collected in 2010 were later analysed. Both of these microelements were determined using ISO 17294-2:2016 method (*16*). The dithiocarbamates were determined using CS_2_, according to CSN EN 12396-2 method (*17*). The potentially active compounds as residues in hop shoots and soil were determined either by gas chromatography with mass spectrometry (6890 GC with 5975 inert MS and with 6890 GC with 5973 inert MS, respectively; both from Agilent Technologies, Santa Clara, CA, USA) or by liquid chromatography tandem mass spectrometry using Agilent 1260 Infinity HPLC (Agilent Technologies) with API 4000 QTrap LC/MS/MS (AB Sciex-Applied Biosystems MS/MS, Framingham, MA, USA). The analyses used are internal methods developed by and individual property of the National Laboratory for Health, Environment and Food, Maribor (Slovenia). The content of these microelements and the potentially active compounds as residues from pesticides were compared to the data from the literature and legislation.

### Statistical analysis

The mass of the fresh white hop shoots was weighed per plant (replication) in each year for each cultivar (treatment), and samples for moisture detection were taken immediately to calculate dry matter yield. The results were processed with the Statgraphics Centurion XVI software (*18*). Differences among cultivars were detected using Duncan’s multiple tests (p=0.05).

The white hop shoots were extracted in parallel duplicates, and each extract from these two replicates was used for the total phenolic analysis in four dilutions, and in parallel triplicates, using the DPPH assay. Each extract was used as duplicate or triplicate in the FRAP assays. The mean values and standard deviations were calculated from these replicate data.

The experimental data on total phenolics, antioxidant potential and ferric reducing antioxidant power were evaluated statistically using the SAS software v. 8.01 (*19*). The data were tested for normal distributions and analysed according to a general linear model procedure. The statistical model included the main effects of cultivar (C): ‘Aurora’, ‘Celeia’, ‘Dana’, ‘Hallertauer Magnum’ and ‘Savinjski golding’, collection year (Y): 2010, 2011, 2012, and their interaction C×Y:


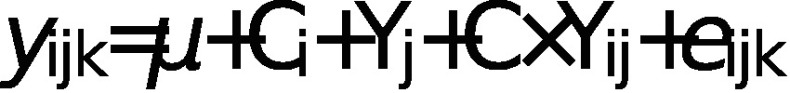


where *y*_ijk_ is an observed value, *μ* is a population mean and *e*_ijk_ is a residual. Interaction C×Y for antioxidant potential of ethanol extracts was not significant, so it was excluded from the model. Mean values were adjusted by the least squares method (LSMEANS) and subsequently compared with Tukey’s test (p<0.05).

## RESULTS AND DISCUSSION

### Moisture content and biomass of the white hop shoots

The white shoots of all five hop cultivars were collected in the first half of April of each of the three years (2009, 2010 and 2011) studied. The water content in the white hop shoots ranged from 72.1 to 88.2% in 2009, from 89.9 to 92.1% in 2010, and from 89.6 to 91.8% in 2011 (data not shown), which was on average larger than reported for green hop shoots (*3*). This is understandable because young hop shoots grow very fast from the buds, and they are full of water, fragile, covered by soil, and white. Later on, their dry mass increases and they become tougher and more green after they emerge from the soil. Interestingly, Ruggeri *et al.* (*3*) did not report any differences in the moisture in the green shoots in two sampling years (~82%) (*3*).

The dry mass of the white hop shoots showed wide variability among the different cultivars in the period from 2009 to 2011 (Table 3). The cultivar *H. lupulus* ‘Aurora’ (3.1 g) had the lowest significant dry mass of white hop shoots per plant, followed by ‘Celeia’ (5.6 g) and ‘Hallertauer Magnum’ (5.9 g). Two cultivars showed the highest significant dry mass of white hop shoots: ‘Dana’ (6.7 g) and ‘Savinjski golding’ (7.1 g). There were also significant differences in the highest dry mass of white hop shoots related to the investigated year. The lowest dry mass of the white hop shoots was observed in the early spring 2011, prior to collection, when precipitation was the lowest (Fig. 1). Recently, Ruggeri *et al.* (*3*) have reported on green shoots of nine hop cultivars. Only one of their cultivars, ‘Hallertauer Magnum’, was the same as in the present study. They determined the dry mass of the green hop shoots per plant (4.26 g in 2013 and 4.01 g in 2014) of each cultivar. Their yield of the green hop shoots per plant differed among the hop cultivars, although not significantly over the 2-year period, except for ‘Hallertauer Aroma’. Therefore, it can be concluded that in their study the dry mass and the yield of green hop shoots per plant depended mainly on the hop cultivar and less on the growing year and weather conditions. In our study, the dry mass of the white hop shoots depended significantly (p<0.005) on both the hop cultivar and on the year of collection (Table 3).

**Table 3:** Mean dry mass of the hop shoots per plant of the different cultivars and sampling years

Parameter	Cultivar					Year			SEM	p-value	
‘Aurora’	‘Celeia’	‘Dana’	‘Hallertauer Magnum’	‘Savinjski golding’	2009	2010	2011	C	Y
*m*(hop shoot)/g	3.1^a^	5.6^b^	6.7^cd^	5.9^bc^	7.1^d^	5.8^B^	7.2^C^	4.0^A^	1.6	<0.005	<0.005

SEM=standard error of the mean, C=effect of cultivar, Y=effect of year of production. Different letters indicate significant differences (p=0.05): ^a-d^=between cultivars, ^A-C^=between years of production

### Total phenolics and antioxidant potential

We carried out preliminary tests to find the most suitable solvent for the total phenolic extraction. Four solvents were tested: acetone, 96% aqueous ethanol (*V*/*V*), 50% aqueous methanol (*V*/*V*), and *N*,*N*-dimethylformamide at different dielectric constants (20.7, 24.55, 32.7 and 36.71). Although 50% (*V*/*V*) aqueous methanol improved the extraction of some phenolics, 96% (*V*/*V*) aqueous ethanol was used here as similar levels of phenolics were extracted, and also to allow closer comparisons with the literature on hop leaves and hop cones (*1*).

Total phenolics and the antioxidant potential according to the FRAP assay in the five cultivars and three years (2010-2012) are given in Table 4. In these white hop shoots, total phenolics as CAE on dry mass basis ranged from 0.576 to 1.790 mg/g. The levels were significantly affected by cultivar (C) (p<0.001), year of production (Y) (p<0.016) and interaction C*×*Y (p<0.001). The highest total phenolics were seen in the extract of *H. lupulus* ‘Dana’ in 2010, and the lowest in the extract of *H. lupulus* ‘Aurora’ and ‘Celeia’ in 2010, ‘Dana’ in 2012, and ‘Hallertauer Magnum’ in each of the three years. In the antioxidant assay, on the other hand, these ethanol extracts showed significant differences in the interaction of the cultivar and year of production (p_CxY_<0.001). The m values were generally significantly higher in 2012, with the exception of ‘Hallertauer Magnum’ (Table 4).

**Table 4:** Total phenolics and ferric reducing antioxidant power expressed as slope of straight line (m) of ethanol extracts from the hop shoots of the different cultivars and sampling years

		Cultivar						
Parameter	Year	'Aurora'	'Celeia'	'Dana'	'Hallertauer Magnum'	‘Savinjski golding’	SEM	p-value
*w*(total phenolics as CAE)/	2010	0.742^gf^	0.674^gf^	1.790^a^	0.639^gf^	1.156^cd^		p_C_ <0.001
(mg/g)	2011	1.131^cd^	0.857^ef^	1.054^ed^	0.684^gf^	1.482^b^	0.002	P_Y_ <0.016
	2012	0.823^f^	1.290^cb^	0.799^gf^	0.576^g^	1.059^ed^		p_CxY_ <0.001
Ferric reducing	2010	0.0063^c^	0.0063^c^	0.0062^c^	0.0063^c^	0.0063^c^		p_C_ <0.673
antioxidant power as m	2011	0.0060^c^	0.0066^bc^	0.0063^c^	0.0069^bac^	0.0066^bc^	0.0000	P_Y_ <0.003
	2012	0.0069^bac^	0.0070^bac^	0.0078^a^	0.0060^c^	0.0075^ba^		p_CxY_ <0.001

SEM=standard error of the mean, C=effect of cultivar, Y=effect of year. Different letters within parameter indicate significant differences (p=0.05) between groups according to the interaction cultivar*×*year of production

The antioxidant potential of white hop shoot extracts determined with DPPH assay is expressed as the IC_50_. The higher the IC_50_, the lower the radical scavenging potential (Table 5). The radical scavenging potential varied significantly among the cultivars (Table 5); however, the differences between the years did not reach significance (p>0.05). Significantly lower radical scavenging potential of ‘Hallertauer Magnum’ and the highest of the extract of ‘Savinjski golding’ and ‘Dana’ was observed.

**Table 5:** Antioxidant potential (IC_50_) of ethanol extracts from the hop shoots of the different cultivars and sampling years

Parameter	Cultivar					Year			SEM	p-value	
‘Aurora’	‘Celeia’	‘Dana’	‘Hallertauer Magnum’	‘Savinjski golding’	2010	2011	2012	C	Y
(IC_50_ as CAE)*/*µg	13.231^bc^	13.797^b^	11.850^c^	15.925^a^	11.700^c^	13.792	13.044	13.065	0.431	0.007	0.647

SEM=standard error of the mean, C=effect of cultivar, Y=effect of year of production. Different letters indicate significant differences (p=0.05) between cultivars

Total phenolics, radical scavenging potential, and ferric reducing power are not always correlated. Correlation among these parameters depends on the structure of the individual phenolics, their redox potentials, and the assay conditions (*e.g.* used solvent or pH value) (*20*). First, for the total phenolic determination, the Folin-Ciocalteu method was used. This method is based on the reduction of the phosphomolybdates and phosphotungstic acid in the reagent with electrons from the compounds with certain redox potential in the extract. Other compounds can also be extracted with ethanol (*e.g.* reducing carbohydrates), and their presence reflects in a higher total phenolic content. Next, for the DPPH^•^ scavenging potential, the ability to donate an electron and/or a hydrogen atom of the compounds is important, and this does not always correlate with the redox potential of the phenolics in the extract (*20*). Some studies have suggested that the individual phenolics can have greater influence on the antioxidant potential than total phenolics, as reported by Abramovič *et al.* (*20*).

In a recently published study where methanol extracts were prepared from young shoots of wild hop collected from four different locations in northern Italy, glycosides of two major flavonols were identified and quantified (*5*). The authors analysed the levels of quercetin and kaempferol glycosides (mainly glucosides and galactosides), using HPLC-UV/DAD, and reported their total range on fresh mass basis from 0.517 to 2.7 mg/g.

The present data for total phenolic content and antioxidant potential of these hop shoots were also compared to those reported for some other vegetables (Table 6 (*1*, *21*)). These data were mainly obtained from the phenol explorer database (*22*), and the data for hop leaves and hop cones are from our previously published study (*1*). The comparisons among these samples revealed that white hop shoots are better antioxidants than hop cones and hop leaves. In these studies, total phenolic content depended not only on the analysed vegetable, but also on the cultivar, pedoclimatic conditions and time of the year of the vegetable collection. Different studies have used different solvents for extraction of phenolics, even from the same vegetable. These reports included 80% (*V*/*V*) aqueous ethanol (*17*, *18*), acetone (*21*, *23*), acetone/5% perchloric acid (80:20; *V*/*V*) (*24*), and ethanol/acetone/water/acetic acid (40:40:20:0.1 V/V) (*25*), compared to the 96% ethanol used in this study. Total phenolics are also expressed in different units, depending on the standard compound used in the assay, although those given in Table 6 were all in mg gallic acid equivalents (GAE) per 100 g fresh mass. The antioxidant potential of different vegetables in Table 6 is also expressed in different units, so the values are difficult to compare.

**Table 6:** Total phenolics and antioxidant potential of hop shoots in comparison with some other raw vegetables

Plant sample	*w*(total phenolics as GAE)/(mg per 100 g)	IC_50_/(mg GAE or µmol TE per 100 g)	Reference
	Mean/range	Mean/range	
Hop shoots			Present study
‘Aurora’, 2010	28.4±43.4	0.014^1^	
‘Hallertauer Magnum’, 2010	22.3±26.1	0.015^1^	
Hop cones			(*1*)
‘Aurora’, 2010	2.9±0.0	(3.8±0.0)^1^	
‘Hallertauer Magnum’, 2010	2.8±0.1	(3.9±0.0)^1^	
Hop leaves			(*1*)
‘Aurora’, 2010	0.8±0.0	(6.4±0.0)^1^	
‘Hallertauer Magnum’, 2010	0.5±0.0	(7.3±0.1)^1^	
Asparagus	14.2-141.0	(1288±130)^2^	(*22*)
Broccoli	25.0-337.0	(3529±353)^2^	
Cauliflower	10.4-274.0	(925±90)^2^	
Cabbage green	52.5-224.0	(2050±21)^2^	
Onion red	81.5-126.0	(1521±138)^2^	(*23*)
Spinach	32.5-483.5	(2732±287)^2^	

^1^GAE=gallic acid equivalents, ^2^TE=Trolox equivalents

### Copper, zinc and active residues from pesticides

One objection against using hop shoots for culinary purposes is a belief that they might contain heavy metals and residues of pesticides as a consequence of their use at hop-growing locations in previous years. For this purpose, in the spring of 2010, both the white hop shoots of *H. lupulus* ‘Dana’ and the soil in which they were grown were collected. This cultivar had the highest total phenolic content and the lowest IC_50_ (*i.e.* the highest antioxidant potential). Its shoots contained copper on fresh mass basis only 2.3 mg/kg (determined with ISO 14235:1998 method (*11*); data not shown). There is no specific copper threshold in hop shoots described in the literature. On the other hand, the mean copper content in other plant tissues on dry mass basis is 10 mg/kg (*26*).

However, the samples of the soil contained 100 mg/kg copper on dry mass basis (determined with ISO 17294-2:2016 method (*16*); data not shown). This value just reaches the warning value for copper in soil according to legislation (*27*), yet it is still well below the critical value (300 mg/kg). The normal range of copper in loamy and clayey types of soil is 25 to 60 mg/kg dm (*27*). Strumpf *et al.* (*28*) investigated hop-growing locations in Germany and reported that the history of hop-growing has a high impact on the copper content in the soil (*27*). The hop fields used for up to 10 years had on average <60 mg/kg copper, fields with hop production for 10 to 50 years had >80 mg/kg copper, and fields where hop-growing has been going on for >50 years had on average >120 mg/kg of copper. They concluded that such contents result from applications from 1924 to 1965, when for plant disease control copper was used up to 60 kg/ha (*28*). Also, an investigation running from 2006 to 2009 of the presence of heavy metals and residues of phytopharmaceutical products in the soil of randomly chosen hop-growing locations in Slovenia (from 15 to 84 soil samples annually with samples taken after harvest) reported relatively high amounts of copper in the majority of the hop-growing locations (*29*). The amount of copper in the analysed soil samples was on average 77.1 mg/kg (minimum 20.6 mg/kg, maximum 177.0 mg/kg). As indicated above, this is a consequence of the procedures used in past decades. Indeed, hop production in Slovenia has 100 years of tradition, hop plants are perennial, copper is very persistent in the environment, and phytopharmaceuticals with copper have been used against downy mildew (*Pseudoperonospora humuli*) for more than 30 years. At present, only up to 3 kg/ha copper *per annum* is allowed to be applied in hop-growing locations, with spraying limited to twice *per annum*. For the last two decades, for hop protection the majority of copper fungicides have been used against secondary infections of downy mildew, based on copper hydroxide and dicopper chloride trihydroxide, for which the withholding period is 14 days.

Although the copper content in the soil where *H. lupulus* ‘Dana’ is grown reached the warning limit, the copper content in the fresh white hop shoots that grew in this soil remained very low (*i.e.* 2.3 mg/kg). These white hop shoots grow very fast from the root system in spring, and thus it appears that they do not accumulate either copper or zinc. In addition, they are harvested early in spring, before any phytopharmaceuticals are used.

According to Bergmann (*30*), plants contain zinc on fresh mass basis between 25 and 150 mg/kg. The white hop shoots of *H. lupulus* ‘Dana’ had much less zinc (4 mg/kg), while the soil from the same hop field had 180 mg/kg, which is also below the limit of 200 mg/kg on soil dry mass basis.

In Slovenia, less than 20 active ingredients are allowed to be used in hop production (*31*). However, for the analysis of potentially active ingredients in the soil and hop shoots that might represent residuals from pesticides or be considered as residues from their use in previous years, we analysed the full list of 182 compounds; these data are given in Table S1. These compounds were all measured below the limits of detection (which for the majority is <0.02 mg/kg) in both the white hop shoots (182 compounds) and the soil samples (180 compounds), except for imidacloprid in the soil (which was also very low, *i.e.* 0.024 mg/kg, and only just above the limit of detection). These data are in agreement with those of Simončič *et al.* (*29*), who reported some residues of phytopharmaceuticals in only a few samples of the soil, and even then only at low mass fractions (up to 0.008 mg/kg), with no active pesticide ingredients from hop-growing reported in the groundwater. The compounds that were detected in this study in the white hop shoots but not in the soil were dithiocarbamates and malaoxon.

The data from these analyses show that there is no concern for the use of these white hop shoots for human consumption. Indeed, the majority (95%) of the hop yield produced in Slovenia is sold to the markets of the EU, America and Japan, where the rules about phytopharmaceutical use are very strict. It is estimated that 100% of hops in Slovenia are produced according to the guidelines of integrated production, with plant protection targeted with optimal phytopharmaceutical use; otherwise, the yield cannot be sold to those markets (*31*). Plant protection follows the instructions of the IHPS, and is based on pest and disease forecasting services (*32*). On average, 19.6 kg active ingredients of phytopharmaceuticals per ha of hop field were used in 2009 and 2010 (24.5 kg/ha in vineyards). The plant protection runs from the beginning of April to the end of August, and is based on five or six applications (April-May, beginning of June, end of June, mid-July, end of July and mid-August), which includes mixed use of fungicides, insecticides and acaricides. Dates of applications depend on a cultivar resistance and maturing times, as well as on the appearance of pests and diseases (*31*).

## CONCLUSIONS

Biomass, total phenolic content, antioxidant activity, microelements and the presence of pesticide residues in white hop shoots of five hop cultivars were analysed. The dry mass of these hop shoots was highly variable among the individual plants within each of the five cultivars, and significantly depended on the cultivar and year (defined in terms of the weather conditions).

Total phenolics and ferric reducing antioxidant power were also significantly affected by cultivar and year, but radical scavenging antioxidant potential varied according to cultivar only. White hop shoots were better antioxidants than hop cones and leaves. In the future, a profile of the phenolics present in the white hop shoots of different cultivars should be investigated.

The hop shoots of *Humulus lupulus* ‘Dana’ collected in 2010 contained very low levels of copper and zinc. This was also the case for all 182 potentially active compounds from pesticides measured in the soil of the *H. lupulus* ‘Dana’ growing location. Although the copper content in the soil was relatively high, this was not reflected on the white hop shoots, where it remained low. The contents of all potentially active compounds were below the limits of detection or slightly over it, *i.e.* far below the permitted values for residues in plant parts intended for human consumption.

## References

[1] Abram V Čeh B Vidmar M Hercezi M Lazić N Bucik V A comparison of antioxidant and antimicrobial activity between hop leaves and hop cones. Ind Crops Prod. 2015;64:124–34. 10.1016/j.indcrop.2014.11.008

[2] Gerhäuser C Beer constituents as potential cancer chemopreventive agents. Eur J Cancer. 2005;41(13):1941–54. 10.1016/j.ejca.2005.04.012 15953717

[3] Ruggeri R Loreti P Rossini F Exploring the potential of hop as a dual purpose crop in the Mediterranean environment: Shoot and cone yield from nine commercial cultivars. Eur J Agron. 2018;93:11–7. 10.1016/j.eja.2017.10.011

[4] CICH – IHB – IHGC International Hop Growers’ Convention (IHGC). Žalec, Slovenia; 2019. Available from: http://www.hmelj-giz.si/ihgc/.

[5] Maietti A Brighenti V Bonetti G Tedeschi P Prencipe FP Benvenuti S Metabolite profiling of flavonols and *in vitro* antioxidant activity of young shoots of wild *Humulus lupulus* L. (hop). J Pharm Biomed Anal. 2017;142:28–34. 10.1016/j.jpba.2017.04.043 28494336

[6] Korber F, Vodušek S. The legend of noble aroma: [Styrian hops]. IHPS: Žalec, Slovenia; 2011 (in Slovene).

[7] Smith KE, Mullins CE. Soil analysis: Physical methods. New York, NY, USA: Marcel Dekker Inc; 1991.

[8] Egner H Riehm H Domingo WR Investigations on the chemical soil analysis as a basis for the assessment of the nutrient status of soils. II. Chemical extraction methods for phosphorus and potassium determination. K Lantbruks Ann. 1960;26:199–215. [in German]

[9] Mihelič R, Čop J, Jakše M, Štampar F, Majer D, Tojnko S, et al. Guidelines for professionally guided fertilization. Ministry of agriculture, forestry and food, Ljubljana, Slovenia: 2010. p. 182 (in Slovene). Available from: https://www.gov.si/assets/ministrstva/MKGP/DOKUMENTI/KMETIJSTVO/RASTLINSKA-PRIDELAVA/GNOJILA/smernice-za-gnojenje_2011.pdf.

[10] Houba VJG Temminghoff EJM Gaikhorst GA van Vark W Soil analysis procedures using 0.01 M calcium chloride as extraction reagent. Commun Soil Sci Plant Anal. 2000;31(9-10):1299–396. 10.1080/00103620009370514

[11] ISO 14235:1998. Soil quality − Determination of organic carbon by sulfochromic oxidation. Geneva, Switzerland: International Organization for Standardization (ISO); 1998. Available from: https://www.iso.org/standard/23140.html.

[12] SIST EN ISO 665:2001. Oilseeds - Determination of moisture and volatile matter content. Ljubljana, Slovenia: Slovenian Institute for Standardization; 2009. Available from: http://ecommerce.sist.si/catalog/project.aspx?id=1729980f-7bbe-4d9f-8d58-e1cf34c05eef.

[13] Singleton VL Rossi JA Colorimetry of total phenolics with phosphomolybdic-phosphotungistic acid reagent. Am J Enol Vitic. 1965;16:144–58.

[14] Brand-Williams W Cuvelier ME Use of a free radical method to evaluate antioxidant activity. Lebensm Wiss Technol. 1995;29:25–30. 10.1016/S0023-6438(95)80008-5

[15] Juntachote T Berghofer E Siebenhandl S Bauer F The antioxidative properties of holy basil and galangal in cooked ground pork. Meat Sci. 2006;72:446–56. 10.1016/j.meatsci.2005.08.009 22061728

[16] ISO 17294-2:2016. Water quality - Application of inductively coupled plasma mass spectrometry (ICP-MS) - Part 2: Determination of selected elements including uranium isotopes. Geneva, Switzerland: International Organization for Standardization (ISO); 2016. Available from: https://www.iso.org/standard/62962.html/.

[17] CSN EN 12396-2. Non-fatty foods - Determination of dithiocarbamate and thiuram disulfide residues - Part 2: Gas chromatographic method. Pilsen, Czech Republic: European Standards; 1998. Available from: https://www.en-standard.eu/csn-en-12396-2-non-fatty-foods-determination-of-dithiocarbamate-and-thiuram-disulfide-residues-part-2-gas-chromatographic-method/.

[18] Statgraphycs Centurion XVI. Statgraphics Technologies, Inc., The Plains, VA, USA; 2017. Available from: http://www.statgraphics.com/download-statgraphics-centurion-xvi.

[19] SAS/STAT^®^ user's guide, v. 8.01, SAS Institute, Inc, Cary, NC, USA; 1999. Available from: https://v8doc.sas.com/sashtml/stat/index.htm.

[20] Abramovič H Abram V Čuk A Čeh B Smole Možina S Vidmar M Antioxidative and antibacterial properties of organically grown thyme (*Thymus* sp.) and basil (*Ocimum basilicum* l.). Turk J Agric For. 2018;42:185–94. 10.3906/tar-1711-82

[21] Chu YF Sun J Wu X Liu RH Antioxidant and antiproliferative activities of common vegetables. J Agric Food Chem. 2002;50(23):6910–6. 10.1021/jf020665f 12405796

[22] Neveu V Perez-Jiménez J Vos F Crespy V du Chaffaut L Mennen L Phenol-Explorer: An online comprehensive database on polyphenol contents in foods. Database (Oxford). 2010;2010:bap024. 10.1093/database/bap024 20428313 PMC2860900

[23] Yang J Meyers KJ Van Der Heide J Liu RH Varietal differences in phenolic content and antioxidant and antiproliferative activities of onions. J Agric Food Chem. 2004;52:6787–93. 10.1021/jf0307144 15506817

[24] Ninfali P Mea G Giorgini S Rocchi M Bacchiocca M Antioxidant capacity of vegetables, spices and dressings relevant to nutrition. Br J Nutr. 2005;93:257–66. 10.1079/BJN20041327 15788119

[25] Howard LR Pandjaitan N Morelock T Gil MI Antioxidant capacity and phenolic content of spinach as affected by genetics and growing season. J Agric Food Chem. 2002;50(21):5891–6. 10.1021/jf020507o 12358455

[26] Yruela I Copper in plants. Braz J Plant Physiol. 2005;17:145–56. 10.1590/S1677-04202005000100012

[27] Alloway BJ. Micronutrients and crop production: An introduction. In: Alloway BJ, editor. Micronutrient deficiencies in global crop production. Dordrecht, The Netherlands: Springer; 2008. pp. 1–39. https://doi.org/10.1007/978-1-4020-6860-7_1

[28] Strumpf T Engelhard B Weihrauch F Riepert F Steindl A Survey of total copper levels in organic and conventionally farmed soil. Part 2: Total contents in soils of German hop growing areas. J Kultpflanzen. 2011;63(5):144–55. [in German]

[29] Simončič A, Sušin J, Baša-Česnik H, Žnidaršič-Pongrac V, Velikonja Bolta Š, Gregorčič A. The influence of hop protection from pests and diseases on the occurrence of plant protection product residues in soils and groundwater in Slovenia. In: Proceedings of the 9th Slovenian Conference on Plant Protection with International Participation; 2009 May 4-5, Nova Gorica, Slovenia; 2009. p. 59 (in Slovene). Available from: http://dvrs.bf.uni-lj.si/spvr/2009/09simoncic_09.pdf.

[30] Bergmann W. Nutritional disorders of plants: Developments, visual and analytical diagnosis. Jena, Germany: Gustav Fischer Verlag; 1992.

[31] Urek G, Knapič M, Zemljič Urbančič M, Škerlavaj V, Simočič A, Persolja J, et al. The use of pesticides and identification of possible solutions for their rational use in Slovenia. Ljubljana, Slovenia: Kmetijski Inštitut Slovenije; 2012 (in Slovene). Available from: https://www.kis.si/f/docs/Publikacije/Raba_FFS.pdf.

[32] Slovenian Institute of Hop Research and Brewing. Žalec, Slovenia; 2019. Available from: http://www.ihps.si/en/about-the-institute/slovenian-institute-hop-research-brewing-shirb/.

